# The oral immune and inflammatory network under circadian clock control

**DOI:** 10.3389/fimmu.2026.1894302

**Published:** 2026-09-07

**Authors:** Xuping Sun, Xiaoxue Ma, Yunjing Ma, Xuezhen Wang, Xiaoguang Li, Mei Ji

**Affiliations:** 1 Department of Stomatology, Shandong Provincial Hospital Affiliated to Shandong First Medical University, Jinan, Shandong, China; 2 Shandong First Medical University, Jinan, Shandong, China; 3 Department of Stomatology, Shizhong District People’s Hospital of Jinan, Jinan, Shandong, China

**Keywords:** circadian rhythm, clock genes, immune and inflammatory diseases, oral microbiome, oral tissues

## Abstract

Circadian rhythms are essential regulators of immune homeostasis, coordinating immune cell functions, inflammatory responses, and tissue repair. Although extensive studies have revealed the importance of circadian regulation in systemic immunity, most mechanistic insights are derived from peripheral immune systems and non-oral tissues. The oral cavity is a unique immune interface continuously exposed to complex microbial communities; however, the role of circadian regulation in oral immune homeostasis and disease development remains poorly understood. This review summarizes the mechanisms by which circadian clocks regulate oral immunity, focusing on immune cell functions, inflammatory responsiveness, tissue homeostasis, and interactions between circadian clock components and inflammatory signaling networks. We further discuss the emerging temporal dynamics of the oral microbiome and the potential effects of host circadian rhythms, feeding schedules, and chrono-nutrition on microbial ecology and immune balance. Current evidence linking circadian disruption with oral diseases, including periodontitis, dental caries, apical periodontitis, Sjögren’s syndrome, temporomandibular joint inflammatory disorders, and oral squamous cell carcinoma, is also reviewed, with emphasis on distinguishing direct oral evidence from extrapolated mechanisms. Finally, we highlight circadian-based therapeutic strategies and existing limitations, underscoring the need for longitudinal studies, multi-omics approaches, and personalized circadian interventions in oral medicine.

## 1 Introduction

The oral cavity is an important interface continuously exposed to the external environment and serves as a key ecosystem maintaining long-term coexistence between the host and microorganisms (1). Unlike relatively enclosed tissues, the oral cavity is constantly challenged by complex exogenous microbial stimuli, including bacterial, fungal, and viral communities (2), while being influenced by periodic environmental factors such as food intake, water consumption, salivary secretion, and oral behaviors (3). Therefore, maintaining oral homeostasis requires not only effective defense against potential pathogenic microorganisms but also precise regulation of commensal microbes to prevent excessive inflammatory responses and tissue damage (4).

The oral immune system possesses unique characteristics. On the one hand, the oral mucosa, periodontal tissues, and salivary glands collectively form multilayered defense barriers that restrict microbial invasion through epithelial barriers, antimicrobial molecule secretion, and innate and adaptive immune responses (5, 6). On the other hand, the oral cavity is continuously exposed to abundant microbial stimuli, requiring the immune system to maintain a dynamic balance between immune tolerance and inflammatory defense (7). Immune cells, including macrophages, neutrophils, dendritic cells, and T/B lymphocytes, continuously participate in oral microenvironment surveillance and inflammatory regulation, making the oral cavity an important model for studying host–microbiome–immune interactions (5).

**Figure 1:**
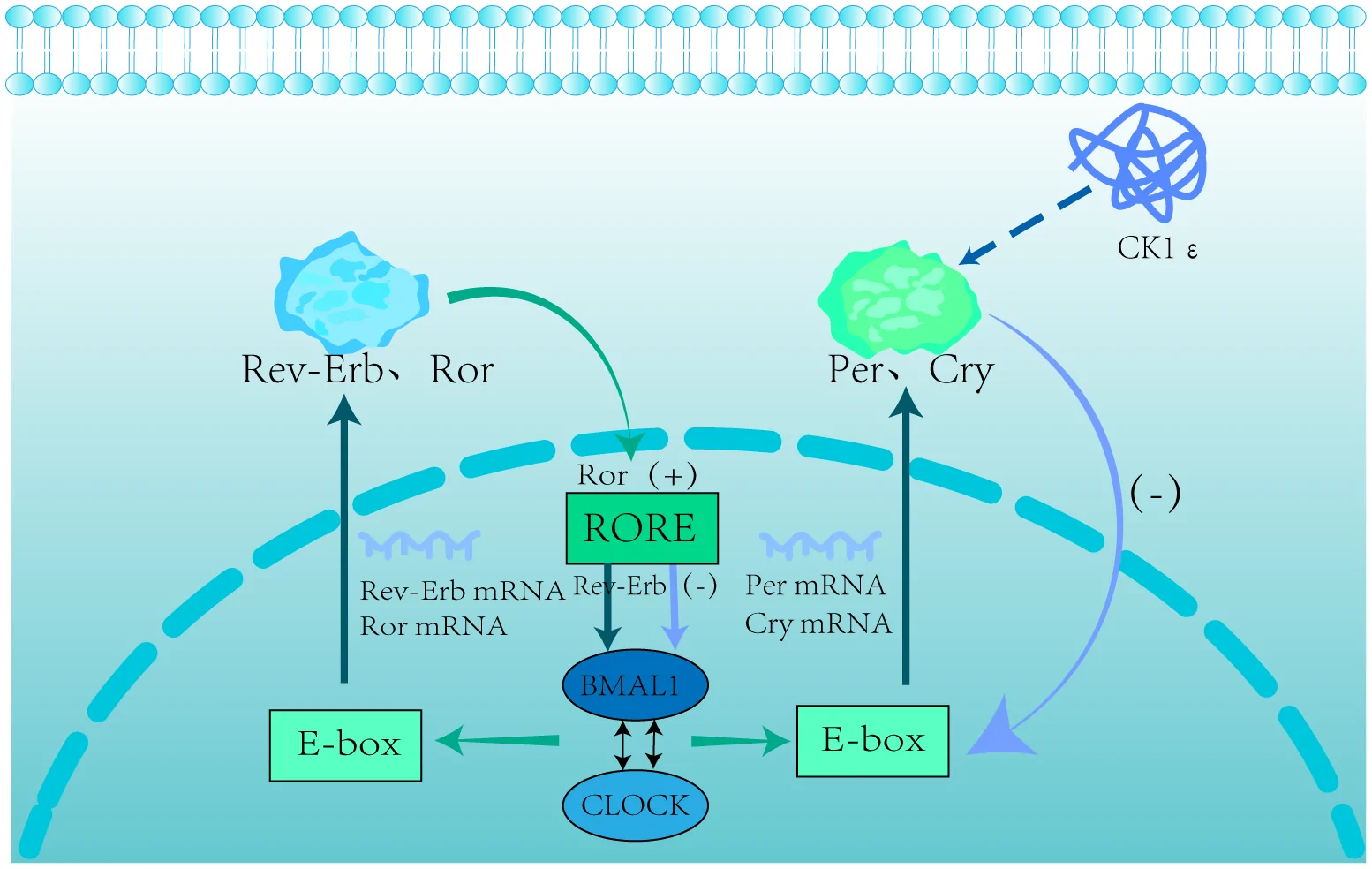
Transcriptional regulation network of core clock genes. Heterodimers formed by CLOCK and BMAL1 bind to the E-box cis-acting elements of target genes, thereby activating the transcription of Per, Cry, Ror and Rev-Erb family genes, which further generate Per, Cry, Ror and Rev-Erb mRNAs respectively. CK1ϵ is involved in the post-translational modification process of the circadian clock regulatory pathway. As a positive regulator (+), ROR exerts an activating effect on downstream gene expression, while REV-ERB acts as a negative regulator (-) to inhibit related gene transcription. The antagonistic interaction between ROR and REV-ERB, together with the CLOCK/BMAL1-mediated transcriptional activation, constitutes a conserved transcription-translation feedback loop (TTFL), which is essential for maintaining the steady-state of circadian rhythmic regulation in mammals.

Circadian rhythm refers to an endogenous and heritable biological rhythm that evolved as an adaptation to the periodic changes in light and temperature caused by the Earth’s rotation, with an approximate 24-hour cycle (8). This rhythmic system can maintain stable oscillations even under constant environmental conditions (such as continuous darkness), demonstrating its intrinsic autonomy (9). In mammals, the circadian system consists of a central pacemaker located in the suprachiasmatic nucleus (SCN) of the hypothalamus and peripheral oscillators widely distributed in almost all organs and tissues (10). These biological clocks are driven by transcriptional–translational feedback loops (TTFLs), involving a series of core clock genes, including Clock, Bmal1, Per, and Cry (10). At the molecular level, BMAL1 and CLOCK proteins form heterodimers in the nucleus and bind to E-box elements within the promoter regions of target genes Per and Cry to activate transcription (11). The resulting mRNAs are translated into proteins in the cytoplasm, which subsequently interact with kinase CK1ϵ and undergo phosphorylation, facilitating their nuclear translocation (12). These proteins then inhibit BMAL1/CLOCK activity through competitive binding to E-box sequences, thereby establishing a negative feedback loop that maintains circadian oscillations (12). In addition, the REV-ERB and ROR families finely regulate this feedback loop by acting as transcriptional repressors and activators, respectively, ensuring the orderly expression of downstream clock-controlled genes and maintaining complex physiological rhythmic processes (13, 14) (**Figure 1**).

In recent years, increasing evidence has suggested that temporal regulation represents an important dimension in maintaining oral dynamic homeostasis (3, 15). The circadian system not only regulates sleep, metabolism, and endocrine processes but also participates in immune regulation and tissue homeostasis (16, 17). Oral tissues may not simply passively receive systemic circadian signals but may possess autonomous local temporal regulatory mechanisms. Previous studies have suggested that salivary glands, oral epithelium, and periodontal tissues express core clock genes, including *Clock*, *Bmal1*, *Per*, and *Cry* family members, and exhibit periodic expression patterns (18, 19). These findings suggest that the oral cavity may contain a temporal regulatory network involving local circadian clocks, immune functions, and microbial ecological environments. Dynamic interactions among local biological clocks, immune cell functions, microbial ecology, and environmental time cues may collectively determine oral immune homeostasis and disease susceptibility. Within this framework, the development of oral diseases may not only result from microbial dysbiosis or immune dysfunction but may also reflect disruption of temporal coordination within the host–microbiome system (3).

This review systematically discusses the regulatory roles of circadian rhythms in oral immune homeostasis, including current limitations of research evidence, the potential contributions of oral microbial rhythms and chrono-nutrition, and future therapeutic strategies based on circadian regulation in oral medicine.

## 2 Local peripheral circadian clock systems in the oral cavity establish the basis for circadian immune regulation

### 2.1 Salivary glands

The saliva in the mouth is mainly produced by three major salivary glands - the submandibular gland, the parotid gland, and the sublingual gland - as well as many small submucosal salivary glands (20). The core clock genes *Bmal1*, *Clock*, *Per1*, *Per2*, and *Cry1* are expressed in the submandibular glands, parotid glands, and sublingual glands of rats and mice, and are mainly located in the nuclei of serous acinar cells and striated duct epithelial cells (21, 22). Among them, the expression levels of *Clock* and *Per2* is particularly significant. Strong positive expressions of clock proteins such as PER1 have also been observed within the mucous acinar cells and striated ducts of human submandibular glands (21, 22). The biosynthetic levels of these genes show a stable 24-hour oscillation rhythm. Under light/dark (LD) conditions, *Bmal1* and *Clock* mRNA reach their peak expression at ZT0 (the start of light), while *Per1* and *Per2* have the highest expression at ZT12 (the start of darkness) (22, 23). This oscillation can still be maintained in continuous darkness (DD), with only a slight reduction in amplitude, confirming the existence of an autonomous peripheral biological clock in salivary glands (22, 23). The clock genes regulate the expression of downstream target genes by directly binding to the E-box elements in the promoter of these genes. Among them, aquaporin 5 (AQP5) and calcium-activated chloride channel protein ANO1, as key molecules for water and ion transport in saliva secretion, have their mRNA and protein expressions regulated by the clock genes and exhibit rhythmicity (21, 23). Moreover, in the DD condition, the peak expression is 6 hours earlier than in the LD condition (23). Deletion of the clock gene or damage to the suprachiasmatic nucleus (SCN) significantly reduces the rhythmic expression of *Aqp5*, *Ano1*, and *polymeric immunoglobulin receptor (pIgR)*, suggesting that the central clock (SCN) and peripheral clock coordinate to regulate the function of salivary glands (23, 24). Saliva not only contributes to lubrication and digestion but also contains abundant immune-related molecules, including immunoglobulins, antimicrobial proteins, and inflammatory mediators. Therefore, local circadian clocks in salivary glands may participate in maintaining oral immune homeostasis by regulating secretory functions and the release of immune-related factors.

### 2.2 Oral mucosa

The oral mucosa serves as the body’s first line of defense, consisting of a physical barrier and a microbial immune barrier involved in maintaining internal environmental homeostasis (25). Alterations in mucosal structure and barrier function can lead to oral lesions as well as systemic diseases (25). In the human oral mucosa, the core clock genes *PER1*, *CRY1*, and *BMAL1* all exhibit clear circadian rhythms (26). *PER1* peaks in expression in the early morning, *CRY1* peaks in the evening, and *BMAL1* is highly expressed at night, while the expression of *CLOCK* and *TIM* does not show significant rhythmic fluctuations (26). The basal cell population of the mouse tongue epithelium exhibits a strong proliferative rhythm, with approximately 54% of basal cells dividing daily (27). Mitotic activity is highly synchronized, peaking around 1:00 PM, and the peak of DNA synthesis occurs 9–12 hours apart from the mitotic peak, forming a typical circadian proliferation cycle (27). This proliferative rhythm is strongly associated with the rhythmic expression of clock genes (26). The peak expression of *PER1* coincides with the timing of the G1 phase marker (p53), while thymidylate synthase, a key enzyme of the S phase, reaches its peak activity in the afternoon, which is highly consistent with the cell cycle progression regulated by clock genes (26). At the cellular level, there are significant circadian differences in the populations of stem cells, progenitor cells, and differentiated cells in the mouse tongue epithelium (28). Progenitor cells are most abundant at ZT0 (beginning of light), whereas non-keratinized mucosal epithelial cells become the dominant population at ZT12 (beginning of darkness) (28). This change depends on clock gene-mediated regulation of the cell cycle (28). In addition, there is individual heterogeneity in the expression of clock genes in the oral epithelium (29). The peak expression of *PER1* and *PER3* gradually delays with age, while the periodic expression of the cell cycle regulatory gene *WEE1* is consistently present in healthy populations, providing a molecular basis for the rhythmic repair of damage in oral tissues (29).

### 2.3 Periodontal tissues

The structural makeup of periodontal tissue encompasses root cementum, alveolar bone, gingiva, and periodontal ligament. clock genes show significant circadian rhythmic expression in periodontal tissues, including the periodontal ligament, alveolar bone, and gingival tissue. After human periodontal ligament fibroblasts (PDLF) are synchronized with dexamethasone, the core clock genes *BMAL1*, *CLOCK*, *PER1*, and *PER2* can exhibit a stable 24-hour oscillation rhythm, consistent with the key characteristics of the transcription-translation feedback loop (30). This rhythmic expression is closely related to periodontal tissue remodeling (30). When *BMAL1* expression peaks, it can simultaneously upregulate the expression of bone metabolism and matrix synthesis markers such as RANKL, OPG, RUNX2, and COL1A1, reflecting the temporal regulatory role of clock genes in periodontal physiological functions (30). Circadian rhythm disorders can disrupt the homeostasis of periodontal tissues (31). Night shift workers experience circadian rhythm disruptions, leading to reduced saliva flow and abnormal blood sugar metabolism, which in turn increases the risk of periodontal disease and dental caries (31). This is directly related to the dysregulation of clock gene expression in periodontal tissues and decreased repair capacity (31). In addition, clock genes can influence fibroblast migration by regulating actin dynamics, providing a molecular basis for the rhythmic repair of periodontal tissue damage (32). These studies confirm the presence of a fully functional clock gene regulatory network in periodontal tissues, which a vital function in standard physiological modulation, orthodontic tissue remodeling, and the pathological progression of periodontitis, providing theoretical support for the temporal intervention of periodontal diseases. However, direct evidence demonstrating circadian oscillations of specific immune cell populations in periodontal tissues remains limited, highlighting the need for further investigation into the temporal regulation of periodontal immune responses.

## 3 Regulatory mechanisms of the circadian clock in immunity and inflammation

The oral cavity is an important part in contact with the external environment, possessing a complex immune defense system that includes numerous immune cells (33, 34). When immune homeostasis is disturbed and inflammatory pathways are activated, it can lead to chronic oral inflammation (34). It is worth noting that immune cells and inflammatory pathways are regulated by circadian rhythms, which will be discussed in the following sections.

### 3.1 Circadian rhythms of immune cells

The first step of immune surveillance involves the precise recruitment and localization of immune cells across different tissues (35). The circadian system does not simply regulate immune cell abundance but rather determines when immune cells enter the circulation and migrate to target tissues by modulating chemokine signaling, adhesion molecule expression, and cellular migratory capacity (35, 36). Monocytes represent one of the best-characterized examples of circadian regulation of immune cell recruitment (37). Nguyen et al. demonstrated that BMAL1 deficiency disrupts the circadian oscillation of Ly6C^hi inflammatory monocytes, impairing their rhythmic recruitment patterns and resulting in abnormal inflammatory responses (37). These findings indicate that BMAL1 does not merely regulate the magnitude of inflammation but also determines, in a temporal manner, whether inflammatory immune cells are in a recruitment-permissive state (37). Similar regulatory mechanisms have been identified in neutrophils (38). Adrover et al. revealed that neutrophils possess a circadian-controlled “timing mechanism” that regulates their aging process, circulating distribution, and tissue infiltration through rhythmic modulation of CXCR2 and CXCR4 expression, thereby coordinating immune defense and inflammatory responses (38). In addition, adaptive immune cells are also regulated by circadian mechanisms (36). Druzd et al. demonstrated that intrinsic circadian clocks within lymphocytes regulate their lymph node homing capacity and adaptive immune responses, suggesting that temporal organization of immune cell spatial distribution represents a conserved regulatory mechanism shared by both innate and adaptive immunity (36). Collectively, current evidence indicates that the first layer of circadian immune regulation lies in determining when immune cells become available and where they exert their functions (35). This temporal organization is particularly relevant to the oral environment, where immune cells must rapidly respond to microbial challenges while simultaneously maintaining immune tolerance toward commensal microbial communities. However, direct evidence regarding circadian patterns of immune cell recruitment within periodontal tissues or oral mucosa remains extremely limited (5).

Following the arrival of immune cells at target tissues, the circadian system further shapes their activation thresholds, functional responsiveness, and phenotypic states (16, 39). Rather than simply altering immune cell abundance, circadian regulation determines how immune cells integrate environmental stimuli, metabolic conditions, and inflammatory signals to generate appropriate immune outcomes (40, 41). This temporal regulation is particularly evident in macrophages, which function as highly adaptable immune sentinels capable of coordinating external challenges with intracellular metabolic and inflammatory programs (42). Macrophages provide a valuable model for understanding how distinct clock components exert specialized effects on immune regulation (42). BMAL1 participates in metabolic-inflammatory regulation by influencing redox balance and inflammatory responses. thereby preventing excessive inflammatory activation (40). Early et al. demonstrated that BMAL1 regulates IL-1β production in macrophages through an NRF2-dependent pathway, establishing a mechanistic connection between circadian machinery and inflammatory mediator production (40). In contrast, PER proteins participate in shaping inflammatory responses and immune adaptation. PER2 influences antimicrobial activity by modulating metabolic adaptation during infection (43). These observations suggest that individual clock components perform distinct yet complementary functions within the immune regulatory framework: BMAL1 primarily supports cellular homeostasis and metabolic stability, whereas PER proteins facilitate functional adaptation following immune stimulation. Beyond the canonical transcriptional feedback loop, REV-ERBα serves as an important molecular link connecting circadian timing with metabolic regulation and inflammatory control (39). By repressing the expression of inflammation-associated genes, REV-ERBα contributes to the prevention of uncontrolled immune activation and fine-tunes innate immune responses (39). Circadian regulation is not restricted to innate immunity but also extends to adaptive immune functions. In T lymphocytes, intrinsic clock machinery participates in coordinating cellular proliferation, migration patterns, differentiation processes, and cytokine production (36). These findings indicate that circadian mechanisms contribute not only to immediate immune responses but also to the establishment of long-term immune stability. Taken as a whole, current evidence supports the concept that the circadian clock functions as a multilayered temporal framework rather than a simple molecular switch controlling immunity. Through the coordinated actions of diverse clock components, cellular metabolism, inflammatory signaling, and differentiation programs are synchronized, allowing immune responses to be appropriately adjusted according to changing temporal environments.

**Figure 2:**
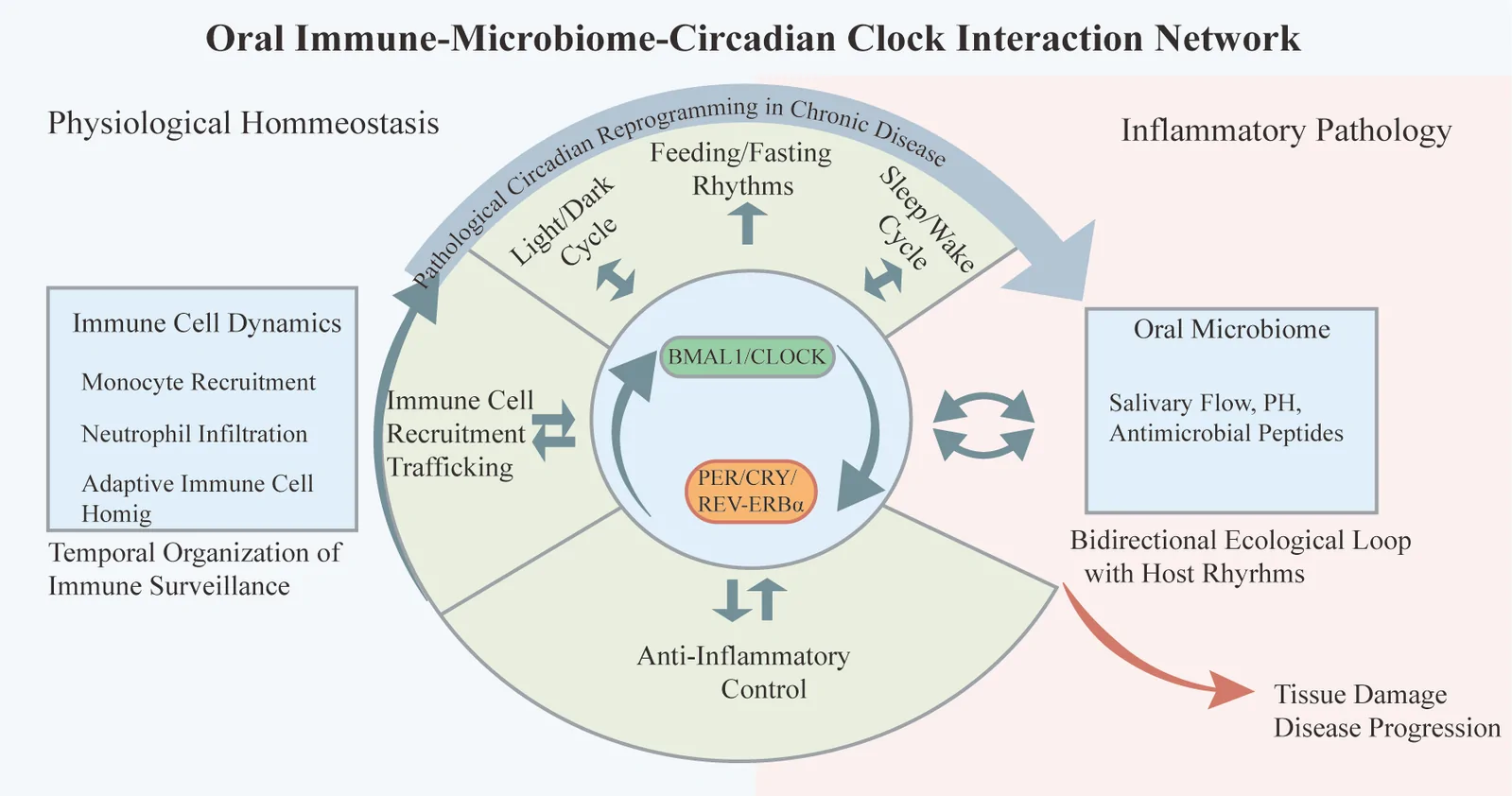
Oral immune-microbiome-circadian clock interaction network. Environmental rhythms entrain the host clock, which orchestrates immune cell trafficking and inflammatory pathways, while reciprocally interacting with the oral microbiome via local cues. Network disruption leads to tissue damage and disease progression.

Effective immune defense requires not only timely activation of immune responses but also appropriate termination of inflammation. The circadian system contributes to this balance by shaping the magnitude and duration of immune activation, thereby coordinating pathogen elimination with tissue preservation (16). The antimicrobial functions of neutrophils and macrophages are both influenced by circadian regulation (38, 40). Through modulation of cellular metabolism, inflammatory mediator production, and migratory dynamics, the molecular clock influences the intensity and persistence of innate immune responses (44). Circadian clock components contribute to host adaptation during infection by shaping immune responses and metabolic programs. These findings suggest that circadian mechanisms not only control inflammatory signaling but also influence the metabolic capacity required for immune cells to execute antimicrobial functions. In addition, REV-ERBα links circadian regulation with innate immune defense by controlling inflammatory gene expression and macrophage responsiveness to microbial stimuli. These studies indicate that circadian regulation does not simply enhance immune activation; rather, it optimizes the timing, magnitude, and duration of immune responses (39). This concept may be particularly relevant to oral tissues. Periodontal tissues, oral mucosa, and dental pulp–periapical tissues undergo continuous renewal and require frequent repair following microbial challenges or tissue injury. Their homeostasis relies on precise coordination between inflammatory responses and regenerative processes. Therefore, circadian disruption may not only promote excessive inflammation but also interfere with subsequent tissue repair and regeneration. However, investigations into circadian regulation of repair processes within oral tissues remain at an early stage. Current mechanistic insights are largely extrapolated from studies in other tissues, and future studies using oral-specific models are required to determine how circadian mechanisms contribute to oral tissue regeneration and disease resolution (**Figure 2**).

### 3.2 Bidirectional regulation between the circadian clock and core inflammatory/immune signaling pathways

The primary control of inflammatory networks by the circadian clock is achieved through rhythmic restriction of transcriptional cascades (39). BMAL1 and REV-ERBα regulate transcriptional accessibility of inflammatory genes and controlling the activity of core inflammatory regulators, thereby establishing time-dependent inhibitory windows during physiological cycles (45). For example, REV-ERBα has been shown to directly repress the transcription of pro-inflammatory genes, including *IL-6* (46), and modulate NF-κB-dependent inflammatory responses through interactions with components of the NF-κB signaling pathway (47). This inhibitory effect is not static but is closely integrated with the activation threshold of the NLRP3 inflammasome. Accumulating evidence indicates that circadian clock components, including CLOCK and BMAL1, influence cellular metabolic states, mitochondrial function, and redox homeostasis (48), thereby affecting ROS production, which serves as an important metabolic signal involved in NLRP3 inflammasome activation (49). Therefore, the coordinated regulation mediated by transcriptional repression through REV-ERBα and metabolic control through BMAL1/CLOCK may enable the immune system to mount appropriately timed responses to microbial challenges while limiting excessive inflammatory damage.

Downstream of inflammatory signaling cascades, the MAPK and JAK–STAT pathways are also subject to precise circadian regulation and exhibit strong context- and disease-state-dependent characteristics (50). Under physiological conditions, circadian clocks establish temporal windows of immune responsiveness, thereby controlling the magnitude and timing of inflammatory activation (51). Rhythmic regulation of cytokine signaling feedback mechanisms contributes to the temporal modulation of inflammatory responses (39, 42). However, persistent inflammatory stimulation can exceed circadian regulatory capacity and reshape immune transcriptional networks (52). Sustained MAPK activation and STAT3 phosphorylation may further influence the core clock machinery (53). Recent evidence demonstrates that STAT3 directly regulates clock gene expression, including *Bmal1*, linking inflammatory signaling with circadian oscillatory properties (53). Disruption of this regulatory network contributes to immune dysregulation and chronic inflammatory diseases) (51, 52).

The precise interaction between the immune system and the circadian clock network may progressively evolve into a state of pathological circadian reprogramming under chronic disease conditions (16, 51). Persistent activation of inflammatory transcription factors, particularly NF-κB and STAT3, can establish a feedback loop between inflammatory signaling and circadian regulation (53, 54). On one hand, excessive inflammatory signaling suppresses the expression of core clock genes, such as *Bmal1*, thereby disrupting normal circadian oscillations (37, 55). On the other hand, inflammation-driven transcriptional regulation and epigenetic remodeling may further alter the functional state of clock proteins and their downstream targets, promoting sustained activation of inflammatory gene programs (56). During this process, REV-ERBα, a key nuclear receptor linking circadian regulation, immune responses, and metabolic homeostasis, plays an important role in maintaining temporal control of inflammatory gene expression (35). Dysregulation of REV-ERBα activity may weaken circadian constraints on inflammatory pathways, thereby facilitating persistent immune activation and impaired restoration of tissue homeostasis (57). In oral pathological settings, including periodontitis and oral squamous cell carcinoma, chronic microbial stimulation, persistent inflammation, and alterations in the tissue microenvironment may further accelerate circadian disruption (16). When endogenous circadian regulation is overwhelmed by sustained inflammatory signaling, immune cells may lose their temporal control of activation thresholds, resulting in impaired inflammatory resolution, accumulated tissue damage, and persistent disease progression.

It should be noted that current mechanistic insights into the interaction between NF-κB/NLRP3/MAPK/JAK–STAT signaling and circadian regulation are largely derived from non-oral tissue models. Moreover, fundamental differences in circadian phase organization between nocturnal rodents and humans should be considered when translating these findings into oral immune contexts. The oral immune microenvironment represents a unique ecological niche characterized by continuous exposure to microbial communities, microbial metabolites, and local inflammatory or mechanical stresses. Therefore, molecular coupling mechanisms identified in systemic inflammatory models may exhibit tissue-specific adaptations within oral tissues. Future studies should move beyond descriptive analyses of pathway interactions and employ oral-specific conditional knockout models, spatial and temporal omics approaches, and human-derived experimental systems to determine which inflammatory cascades are directly governed by intrinsic circadian regulation and which represent secondary circadian remodeling induced by pathological stress (**Figure 2**).

### 3.3 The oral microbiome and circadian rhythms: a bidirectional ecological loop

The oral microbiome is not a static microbial community but rather exhibits dynamic temporal characteristics, with its composition, metabolic activity, and functional outputs fluctuating across the day-night cycle (58). Recent high-frequency saliva sampling studies have demonstrated that the oral microbiota displays significant diurnal variation, with specific microbial taxa and metabolic pathways showing time-dependent oscillatory patterns (59). These rhythmic changes are not solely driven by host circadian clock mechanisms but are also tightly coupled with local oral environmental fluctuations, including salivary secretion, pH variation, redox status, antimicrobial peptide production, and feeding behavior (60, 61).

Disruption of microbial rhythmicity may impair ecological homeostasis and promote a shift toward inflammatory and pathogenic states (58). Emerging evidence suggests that circadian disruption caused by factors such as shift work, sleep deprivation, and social jet lag can alter oral microbial composition and may contribute to increased susceptibility to oral inflammatory diseases (62). However, whether alterations in oral microbial rhythms directly drive inflammatory progression or represent secondary consequences of host circadian disruption remains to be determined.

The temporal dynamics of oral microorganisms are reflected not only in changes in microbial abundance but also in the rhythmic remodeling of metabolic profiles and functional pathways. Host circadian regulation may shape the oral microbial ecosystem through rhythmic modulation of salivary flow, immunoglobulin A (IgA), defensins, and other antimicrobial molecules (61). For example, the physiological reduction in salivary flow during the night decreases oral clearance capacity and alters nutrient availability, oxygen gradients, and ecological competition, thereby influencing the balance between different microbial niches (63).

Conversely, microbial-derived metabolites may reciprocally regulate host immune and circadian networks. Studies from the gut microbiome field have demonstrated that microbial metabolites, including short-chain fatty acids (SCFAs), can influence host immune cell function through signaling pathways such as G protein-coupled receptors (GPCRs) and participate in the regulation of peripheral circadian rhythms (64). However, whether similar mechanisms operate within the oral microbiome remains largely unexplored and requires direct experimental validation.

Despite increasing interest, research on circadian regulation of the oral microbiome remains largely descriptive. Most current evidence is derived from cross-sectional metagenomic or metabolomic analyses, whereas experimental models capable of defining causal interactions between host rhythms and microbial oscillations remain limited. Future studies should integrate chronomics approaches, high-frequency longitudinal sampling, microbial functional validation, and host-specific circadian gene manipulation models to determine which oral microbial populations and metabolites can actively regulate local immune rhythms. Such investigations may reveal novel time-based therapeutic strategies for controlling oral inflammatory diseases through microbiome modulation (**Figure 2**).

## 4 Circadian regulation in oral immune and inflammatory diseases

### 4.1 Periodontitis

Periodontitis is a prevalent chronic inflammatory oral disease characterized by progressive periodontal tissue destruction and alveolar bone loss. Increasing evidence suggests that modern lifestyle factors associated with circadian rhythm disruption (CRD), including insufficient sleep and irregular sleep–wake patterns, may contribute to periodontal disease susceptibility (65). Epidemiological studies in adult populations have demonstrated associations between impaired sleep quality or reduced sleep duration and increased periodontal disease severity, suggesting that circadian disturbance may represent an important environmental modifier of periodontal inflammation (66). However, whether CRD acts as a causal driver of periodontitis progression remains to be established through mechanistic studies.

Experimental studies using ligature-induced periodontitis models have provided initial evidence linking circadian dysfunction to periodontal inflammation. Circadian disruption or alteration of core clock gene expression, particularly *Bmal1*, has been associated with enhanced NF-κB activation, increased oxidative stress responses, including elevated reactive oxygen species (ROS) production, and accelerated periodontal tissue injury (67). However, ligature models rely on acute and intense bacterial accumulation and mechanical injury, which may not fully recapitulate the chronic and slowly progressive nature of human periodontitis, highlighting an important translational gap. More physiologically relevant genetic models have further demonstrated the involvement of the circadian clock in periodontal immune regulation. Loss of *Bmal1* function has been shown to enhance inflammatory responses through increased NF-κB/p65 activation and elevated production of pro-inflammatory cytokines, including IL-1β, IL-6, and TNF-α, thereby promoting periodontal tissue destruction (68). These findings suggest that *Bmal1* serves as an important molecular checkpoint linking circadian regulation with periodontal immune homeostasis.

Beyond classical inflammatory pathways, recent studies indicate that *Bmal1* regulates multiple forms of programmed cell death within periodontal tissues. In gingival fibroblasts, circadian regulation through the REV-ERBα axis has been implicated in controlling inflammatory responses, whereas disruption of *Bmal1* signaling may enhance inflammasome activation and inflammatory cell death (69). In periodontal ligament stem cells (PDLSCs), reduced *Bmal1* expression has been associated with activation of stress-related pathways, impaired osteogenic differentiation, and increased susceptibility to inflammatory damage, thereby compromising periodontal regeneration capacity (70).

Pharmacological targeting of circadian regulators, including agonists (SR8278, SR1078) has shown potential protective effects in experimental inflammatory models (71). However, most current interventions remain focused on individual signaling nodes and do not fully account for the complexity of circadian compensation, tissue-specific regulation, and systemic metabolic interactions.

In addition, other clock-controlled regulators, particularly REV-ERBα (encoded by NR1D1), participate in periodontal inflammatory regulation. Altered REV-ERBα activity may influence osteoclast differentiation and inflammatory bone remodeling through interactions with cytokine signaling networks, including IL-22/STAT3-associated pathways (71). These findings highlight a complex regulatory interface between circadian transcriptional programs and immune-mediated periodontal destruction.

**Figure 3:**
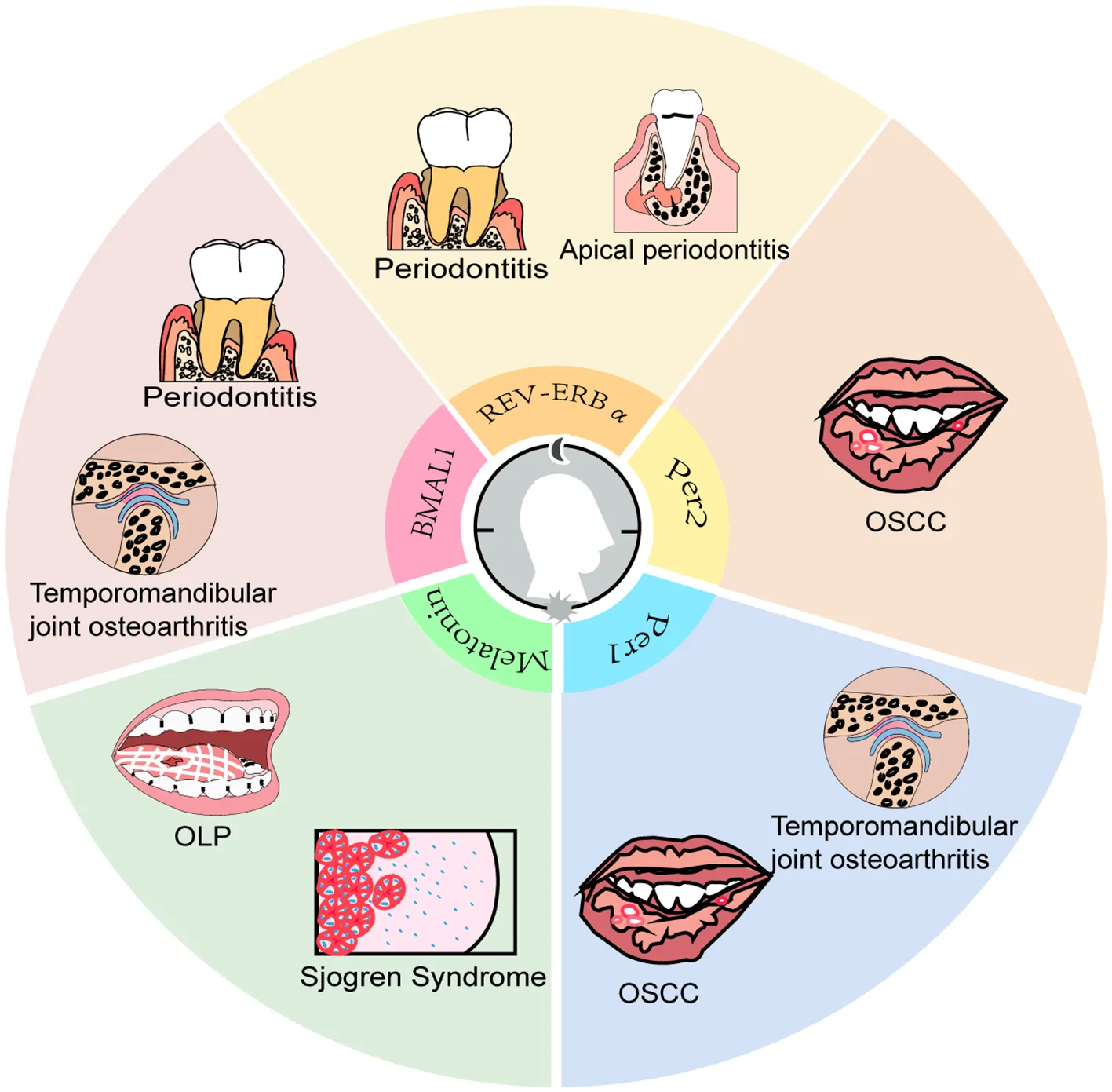
Circadian regulation in oral immune-inflammatory diseases. The circadian clock, composed of core regulators such as BMAL1, REV-ERBα, PER2, and other clock-controlled factors, is involved in the regulation of oral immune homeostasis and inflammatory diseases. Circadian disruption is associated with periodontal diseases, apical periodontitis, OSCC, Sjögren syndrome, OLP, and TMJOA, suggesting a potential role of circadian regulation in oral disease progression and therapeutic intervention.

Collectively, current evidence supports circadian disruption as an important modulator of periodontal inflammation and tissue destruction. However, existing studies remain largely reductionist, focusing predominantly on individual clock genes and linear signaling pathways. The coordinated functions of other core clock components in maintaining periodontal homeostasis remain poorly understood. Future investigations should move beyond single-gene analyses toward an integrated understanding of the periodontal molecular clock network. Such approaches will be essential for translating circadian-based interventions and chronotherapeutic strategies into clinical applications for periodontal disease management (**Figure 3**; **Table 1**).

**Table 1:** Circadian regulation in oral immune and inflammatory diseases.

Disease/pathological condition	Core molecular mechanisms	Experimental models	Limitations
Periodontitis	Inhibition of NF-κB, inhibition of NLRP3/pyroptosis, regulation of PDLSCs via ERK/AP-1	Mouse ligature model; P. gingivalis induction; BMDM cells	Acute models fail to fully recapitulate the complexity of human chronic disease
Apical periodontitis	Buffering bacterial inflammation; mediation of M2 macrophage polarization	E. faecalis-induced rat model	Research is in its early stages; key target cells remain to be confirmed
Oral Lichen Planus	Melatonin-mediated local antioxidant/anti-inflammatory feedback loop	Human tissue biopsies; immunohistochemistry	Evidence is primarily observational; lacks functional validation of causality
Sjögren Syndrome	Regulation of immune balance (Th17/Treg); restoration of rhythmic AQP5 secretion; gut microbiota-butyrate axis	NOD mouse model; human samples	Clinical translation between NOD mouse models and human subtypes remains debated
Temporomandibular Joint Osteoarthritis	NF-κB/MMP13 axis; GSK3β/β-CATENIN; MAPK/ERK-mediated cartilage degradation	Rat UAC model; chondrocyte culture	Lacks human tissue data; requires further exploration of the ecophysiological effects of circadian and ultradian rhythms
Oral Squamous Cell Carcinoma	AKT/mTOR-mediated autophagy inhibition; IKK/NF-κB degradation; PD-L1/immune escape	Cell lines; xenograft models	Over-reliance on simplified *in vitro* models; lacks simulation of the complex tumor microenvironment

### 4.2 Apical periodontitis

Apical Periodontitis (AP) is a very common inflammatory disease in dental clinics, characterized mainly by the resorption of alveolar bone around the apex (72). Similar to the pathological process of periodontitis, clock genes are additionally implicated in the inflammatory regulation of AP. Epidemiological studies have first found that CRD is an independent key factor promoting the acute exacerbation of AP, and the core mechanism of this process is related to the downregulation of the Bmal1 (73). Subsequent animal experiments further confirm that CRD exacerbates inflammation in periapical tissues and alveolar bone resorption by downregulating the expression of the *Bmal1 (*73). Notably, in rats with *Bmal1* gene knockout, the inflammatory response and bone destruction in periapical periodontitis are significantly more severe (73). Interestingly, exogenous supplementation of melatonin could restore the body’s circadian rhythm, stabilize *Bmal1* expression, and effectively alleviate periapical inflammation and bone resorption, offering a novel direction for the adjuvant therapy of periapical periodontitis (73). Experimental studies by Kırmızı et al. also support the therapeutic value of melatonin. They observed that systemic administration of melatonin significantly lowered circulating levels of pro-inflammatory mediators such as TNF-α and IL-1β, as well as inflammation-related enzymes like ALT, AST, and LDH (74). Micro-CT examinations further confirmed that melatonin could reduce the volume and surface area of periapical bone resorption cavities. This finding holds promise for the treatment of human pulp diseases, but further studies are still needed to validate it (74). In addition, regarding refractory apical periodontitis (RAP), the study by Song and colleagues also reveals the regulatory role of the core clock gene *Rev-erbα* (75). In a RAP model induced by enterococcus faecalis, the expression of *Rev-erbα* is significantly downregulated; however, by applying the agonist SR9009 within the root canal, macrophages could be induced to polarize toward the M2 type, enhancing the production of anti-inflammatory cytokines such as IL-10 and TGF-β, while diminishing the release of pro-inflammatory factors, ultimately inhibiting osteoclast activity (75). This finding provides experimental support for the application of circadian rhythm regulation strategies in the treatment of RAP (75). Compared with periodontitis, research into the circadian rhythmicity of apical periodontitis remains in its infancy. Although current experimental data from rodent models and pharmacological agonist administration are encouraging, the specific immune cell subpopulations within apical granulomas that serve as critical targets for circadian regulation remain elusive. Consequently, it is premature to extrapolate these findings directly into clinical protocols. Furthermore, it is imperative to determine whether the therapeutic efficacy of agents such as melatonin or SR9009 is solely attributable to the precise modulation of circadian clock genes, or whether it derives from their broad-spectrum antioxidant or immunomodulatory properties. Future clinical studies must validate whether circadian-based interventions can serve as effective adjuvant strategies to conventional root canal therapy, thereby translating into tangible clinical benefits (**Figure 3**; **Table 1**).

### 4.3 Oral mucosal diseases

Core circadian rhythm genes exhibit functional rhythmic expression in the oral mucosa. In healthy males, *PER1*, *CRY1*, and *BMAL1* in the oral mucosa show peak expression in the early morning, afternoon, and night, respectively, while *CLOCK* and *TIM* do not display rhythmicity (76). Moreover, the peak of *PER1* coincides with the timing of the G_1_ phase cell cycle marker (p53), and the peak of *BMAL1* overlaps with the M phase marker (cyclin B1), suggesting that circadian rhythms help to maintain normal proliferation and repair homeostasis of the oral mucosa by regulating the cell cycle (76). This provides a theoretical basis for the time-dependent administration of chemotherapeutic drugs, such as 5-FU (26). In oral lichen planus (OLP),patients have higher levels of melatonin in their mucosa compared to normal mucosa (77). Therefore, Chaiyarit and colleagues hypothesized that the chronic inflammation of the oral mucosa in OLP patients may induce local melatonin synthesis through the NF-κB signaling pathway, and that melatonin participates in the pathological process by exerting biological functions such as regulating circadian rhythm, anti-inflammation, and immune modulation (77). The immunohistochemical study by Luengtrakoon et al. further confirmed that in OLP patients, the expression levels of the rate-limiting enzyme involved in melatonin biosynthesis, AANAT (Arylalkylamine N-acetyltransferase), melatonin itself, and the melatonin receptor MT1 in inflamed oral mucosa were significantly higher than those in normal oral mucosa (78). This increase was independent of age, suggesting that chronic inflammation may mediate local melatonin biosynthesis through AANAT and enhance its antioxidant, anti-inflammatory, and immunomodulatory effects via MT1 (78). This, in turn, provides a protective function for damaged oral epithelial cells, reducing oxidative stress and inflammatory damage, and may also influence the clinical phenotype of OLP by regulating epithelial cell permeability (78). The aforementioned studies reveal that the circadian rhythm system participates in the maintenance of oral mucosal homeostasis through multiple pathways, including the regulation of core genes, hormone secretion, and downstream signaling pathways. Its disruption or abnormal expression of related molecules is an important pathogenic mechanism of inflammatory diseases of the oral mucosa, providing a solid experimental basis and new ideas for circadian rhythm-targeted diagnosis and treatment of such diseases. However, current research is based on laboratory methods such as tissue biopsy and immunohistochemistry, with limited sample sizes. Further research is warranted to expand the sample size and conduct animal studies as well as clinical trials to verify the specific regulatory mechanisms (**Figure 3**; **Table 1**).

### 4.4 Sjögren syndrome

Sjögren’s syndrome, or sicca syndrome, is a chronic autoimmune epithelial inflammation characterized by lymphocytic infiltration of exocrine glands, especially the salivary and lacrimal glands, leading to progressive impairment of gland function and resulting in dry mouth and dry eyes (79). Studies have confirmed abnormal expression of circadian clock genes in SS (Sjögren Syndrome) patients and NOD (Non-Obese Diabetic) mice, characterized by downregulation of rhythm-suppressing genes such as *Bmal1*, *Cry1*, and *Cry2*, and upregulation of pro-inflammatory rhythm genes, including *Per1*, *Per2*, *Clock*, suggesting that their pathological processes are closely related to circadian rhythm disorders (80). Interestingly, melatonin levels in the serum and saliva of SS patients are also significantly reduced, and salivary melatonin levels are inversely correlated with unstimulated saliva flow, suggesting that endogenous melatonin deficiency may be involved in the pathological process of SS (81). As the core regulatory hormone of the circadian rhythm, melatonin’s protective effects on SS have been well established: on the one hand, melatonin can inhibit the IL-6/STAT3 signaling pathway through receptor-dependent mechanisms (membrane receptors MT1/MT2 and nuclear receptors RORα/RORγ), alleviating inflammatory infiltration, oxidative stress damage, and cell apoptosis in salivary gland tissue, while also regulating the expression and localization of the water channel protein AQP5 (Aquaporin 5) to restore salivary secretion function (82). On the other hand, melatonin can target and regulate immune cell balance, inhibiting the proliferation of pathogenic Th17 cells and CD4^-^CD8^-^ double-negative (DN) T cells and the secretion of inflammatory cytokines (IL-17, IL-6, etc.) through a nuclear receptor-dependent mechanism, while promoting the development of Treg and Th2 cells and the production of anti-inflammatory factors (IL-10, TGF-β), thereby improving the immune disorder state of SS (80, 81). In addition, the short-chain fatty acid butyrate, a metabolite of the gut microbiota, also shows circadian rhythm-related therapeutic potential in SS. In SS model mice, the abundance of butyrate-producing bacteria in the gut is reduced, while exogenous butyrate can induce IL-10-producing B cells (B10 cells) and inhibit IL-17-producing B cells by regulating circadian rhythm-related genes (RORα, NR1D1) (83). At the same time, it modulates the balance of apoptosis and necrosis in salivary gland cells, alleviating glandular inflammation (83). These studies collectively indicate that circadian rhythms are deeply involved in the onset and progression of SS, and they highlight the important role of melatonin, opening a new perspective for SS treatment. The current understanding of circadian disruption in Sjögren’s syndrome (SS) has established a multidimensional regulatory network involving the microbiome, immune cells, and neuroendocrine systems. However, significant challenges remain in translating these findings into clinical applications. Most evidence regarding melatonin-based interventions has been derived from NOD mouse models, and its efficacy, dose–response relationship, and long-term safety across different clinical subtypes of SS remain insufficiently validated. Moreover, whether the protective effects of melatonin are primarily mediated through circadian synchronization or through its broader antioxidant and anti-inflammatory activities, such as regulation of NF-κB and NLRP3 signaling, remains unclear. Future studies should integrate circadian monitoring, multi-omics approaches, and personalized interventions to define optimal treatment windows and develop chronotherapy-based strategies, thereby advancing SS management from conventional immunomodulation toward precision circadian medicine (**Figure 3**; **Table 1**).

### 4.5 Temporomandibular joint osteoarthritis

Temporomandibular joint osteoarthritis (TMJOA) is an important subtype in the classification of temporomandibular joint disorders. The pathological features of TMJOA include progressive cartilage degradation, subchondral bone remodeling, and chronic inflammation of the synovial tissue (84). An increasing number of people are paying attention to the exact pathogenesis and progression of TMJOA. Accumulating recent studies have indicated that clock gene disturbance serves as a critical risk determinant contributing to the occurrence and development of TMJOA, with its core mechanism intimately linked to abnormal rhythmic expression of clock genes and the activation of downstream signal pathways (85). Evidence indicates that in rat mandibular condylar chondrocytes, the genes such as *Per1*, *Per2*, *Clock*, and *Cry1*, as well as the cartilage matrix degradation factor gene *Mmp13*(Matrix Metalloproteinase 13), are all rhythmically expressed (86). Moreover, the expression curves of *Mmp13* and *Per1* are highly synchronized in both phase and amplitude, suggesting a regulatory association between them (86). In the IL-1β-induced *in vitro* TMJOA model, *Per1* expression was significantly increased, accompanied by activation of the NF-κB pathway (increased phosphorylation of P65) (86). Downregulation of *Per1* inhibited *Mmp13* expression and NF-κB pathway activation. Similarly, in the *in vivo* TMJOA model induced by unilateral anterior crossbite (UAC), elevated expression of *Per1*, *Mmp13*, and *P65* was observed, along with reduced cartilage thickness and deterioration of bone parameters (86). This study suggests that *Per1* regulates disease progression through the NF-κB pathway. Subsequent research revealed another association between *Per1* and TMJOA. Researchers, by establishing a rat jet lag model, found that the mandibular condyles of the rats exhibited TMJOA-like lesions, characterized by cartilage degeneration, subchondral bone resorption, and increased modified Mankin scores, with upregulated expression of *Per1* and *Mmp13* during this process (87). In experiments with isolated chondrocytes *in vitro*, upregulation of *Per1* activated the GSK3β/β-CATENIN pathway, upregulating the expression of matrix-degrading enzymes including Mmp13, ADAMTS4, and ADAMTS5, whereas *in vivo* downregulation of *Per1* significantly inhibited the activation of this pathway, alleviating cartilage and bone tissue damage (87). In addition, another clock gene, *Bmal1*, also regulates the expression of cartilage genes in TMJOA through the MAPK/ERK pathway, with IL-6 acting as a key cytokine involved in this regulatory process (88). It is worth noting that TMJOA-related research needs to pay attention to ecological validity. The dynamic changes of circadian and ultradian rhythms (such as dysregulation of the autonomic nervous system’s ultradian cycles) are closely related to TMJ pain, and objectively capturing an individual’s circadian rhythm status is crucial for elucidating the pathogenesis and optimizing intervention strategies (85). In summary, the clock genes *Per1* and *Bmal1* are important factors related to TMJOA, and the involved inflammatory pathways are diverse, but the specific molecular targets within these pathways have not been clearly identified. Although current animal models, including unilateral anterior crossbite (UAC) models and circadian disruption paradigms, have provided evidence linking clock genes with TMJ osteoarthritis (TMJOA) progression, their clinical translation remains limited by insufficient ecological validity. Most mechanistic studies have been conducted in rodent models, while direct evidence regarding the temporal expression patterns of circadian genes in human TMJ tissues remains scarce.

Moreover, rhythmic regulation within joint tissues is not solely determined by the core circadian clock but may also interact with autonomic and ultradian rhythmic processes. However, current studies have largely focused on circadian-scale alterations, leaving the contribution of multi-scale rhythm disruption to TMJ inflammation and pain insufficiently understood. Future investigations should move beyond single clock gene manipulation, such as *Per1* or *Bmal1* knockout models, and integrate molecular clock regulation with clinically relevant chronotherapeutic approaches. Such efforts may facilitate the development of rhythm-based interventions for TMJOA (**Figure 3**; **Table 1**).

### 4.6 Oral squamous cell cancer

The incidence and mortality rates of oral squamous cell carcinoma are among the highest in the world, and its causative factors include smoking, drinking, and chewing betel nut (89, 90). Current research has found that clock genes are abnormally expressed in oral squamous cell cancer (OSCC), suggesting a new carcinogenic risk. *Per1* is significantly downregulated in OSCC tissues and cells, and its loss of expression is closely associated with TNM clinical stage progression and poor prognosis in patients (91). Mechanistically, *Per1* knockdown can inhibit autophagy-mediated apoptosis and promote tumor cell proliferation by activating the AKT/mTOR pathway (91). At the same time, it disrupts the expression balance of multiple genes in the circadian gene network, such as *Per2*, *DEC1*, and *DEC2 (*92), affecting the transcription of downstream inflammation-related oncogenes (e.g., KI-67, MDM2) and tumor suppressor genes (e.g., p53) (93), thus attenuating the immune surveillance against tumor cells. As an important tumor suppressor gene, low level of *Per2* is also linked to the malignant progression of OSCC. On one hand, *Per2* can directly bind to and stabilize Caspase-8 protein, promoting the formation of the Caspase-8/RIPK3/ASC complex, activating PANoptosis inflammatory cell death pathways, and effectively eliminating tumor cells (94); On the other hand, *Per2* binds to HSP90 through its PAS1 domain, reducing the interaction between HSP90 and IKKs, promoting the ubiquitin-mediated degradation of IKKα/β, thereby inhibiting the IKK/NF-κB inflammatory signaling pathway and the nuclear translocation of p65, downregulating the expression of the immune checkpoint molecule PD-L1 (95). Simultaneously, it promotes the infiltration of CD8 T cells into tumor tissues, enhancing T cell-mediated antitumor killing (95). Targeting *Per2* combined with anti-PD-L1 therapy can significantly improve the therapeutic effect in OSCC (95). Studies indicate that the circadian rhythm genes *Per1* and *Per2* form a key immune-inflammatory network regulating the development of OSCC by modulating inflammatory signaling pathways (AKT/mTOR, IKK/NF-κB), inflammation-related cell death (PANoptosis), immune cell functions (CD8 T cells), and tumor immune evasion (PD-L1 expression), providing new molecular targets for targeted therapy and immunotherapy in OSCC. Current studies on circadian regulation in oral squamous cell carcinoma (OSCC) largely rely on cell lines and xenograft tumor models, which may not fully recapitulate the heterogeneity and dynamic rhythmic alterations within the human tumor microenvironment (TME). Although emerging evidence suggests that core clock genes, particularly *Per2*, may regulate immune-related molecules such as PD-L1 and provide a conceptual basis for circadian-based immunotherapy, whether this regulatory mechanism is conserved across different OSCC stages, molecular subtypes, and immune contexts remains unclear. Future studies should move beyond functional characterization of individual clock genes and explore the therapeutic potential of circadian modulation in combination with immune checkpoint inhibitors (ICIs). Integrating longitudinal clinical sampling and circadian profiling will be essential to identify optimal intervention windows and develop chronotherapy-based strategies for improving OSCC treatment outcomes (**Figure 3**; **Table 1**).

## 5 Discussion

Circadian rhythms represent a fundamental biological timing system that maintains physiological homeostasis and coordinates diverse biological processes. In recent years, circadian regulation has increasingly been recognized as a critical temporal dimension linking immune homeostasis, microbial ecosystem stability, and disease progression. However, compared with the extensive advances achieved in metabolism and neuroscience, circadian biology in the oral cavity remains at an early stage of development. Accumulating evidence demonstrates that the circadian system orchestrates immune cell trafficking, inflammatory responsiveness, and tissue repair through an interconnected molecular clock network. Core clock components, including BMAL1, CLOCK, PER/CRY, and REV-ERB, not only sustain intrinsic cellular oscillations but also regulate immune homeostasis by reshaping transcriptional programs, metabolic states, and inflammatory signaling landscapes.

Nevertheless, it is important to recognize that much of the current mechanistic understanding of circadian immune regulation originates from studies of peripheral immune systems, metabolic disorders, and non-oral tissues. The oral cavity represents a highly specialized immune niche characterized by continuous microbial exposure, unique mucosal barrier properties, and complex local immune interactions. Therefore, regulatory mechanisms identified in other tissues cannot be directly extrapolated to oral environments. At present, whether immune cells within periodontal tissues, oral mucosa, salivary glands, and dental pulp/periapical tissues possess intrinsic circadian oscillations, and how these local rhythms contribute to oral disease susceptibility and progression, remain largely unexplored.

Recent advances in oral microbiome research have further expanded the conceptual framework of circadian regulation in oral biology. Traditionally, the oral microbiota has been regarded as a relatively stable ecological community; however, high-frequency temporal sampling approaches have revealed that microbial composition, functional metabolic profiles, and ecological interactions exhibit substantial time-dependent fluctuations. These findings suggest that the oral microbiome possesses inherent temporal organization. Host circadian rhythms, sleep–wake cycles, salivary secretion patterns, and feeding behaviors collectively shape the oral microenvironment, whereas microbial-derived metabolites may reciprocally influence host immune responses and local inflammatory states. Thus, future investigations should move beyond conventional analyses of microbial composition changes and instead focus on the temporal architecture of oral ecosystems, elucidating the dynamic interactions among host rhythms, microbial oscillations, and immune regulation.

Of particular interest, feeding–fasting cycles serve as important entraining signals for peripheral clocks and may influence oral homeostasis by modulating salivary microbial communities, nutrient availability, and metabolic environments. However, the precise contribution of nutritional timing to oral disease development remains poorly understood. Future studies integrating dietary timing records, longitudinal saliva sampling, and multi-omics analyses will be essential to determine how metabolic time cues reshape oral microbial and immune networks.

Although accumulating evidence indicates associations between circadian disruption and various oral diseases, establishing causal relationships remains a major challenge. Sleep disturbances, irregular lifestyles, and social behavioral factors have all been linked to impaired periodontal health; however, whether these factors directly promote disease through disruption of circadian regulation or indirectly influence disease susceptibility through immune, metabolic, and microbial alterations remains unresolved. Addressing this question will require a transition from conventional single-time-point measurements toward dynamic experimental frameworks incorporating longitudinal sampling, time-series transcriptomics, single-cell sequencing, and spatial omics approaches. Such strategies will enable the reconstruction of temporal sequences and causal relationships underlying circadian alterations during oral disease progression.

Furthermore, a more oral-specific framework for circadian immunology needs to be established. Current studies of circadian immune regulation have primarily focused on macrophages, monocytes, and circulating immune cells, whereas the rhythmic properties of periodontal immune populations, oral mucosal immune cells, and salivary immune components remain insufficiently characterized. Integrating oral-specific animal models, human-derived tissue samples, and multidimensional omics platforms will be critical for defining the functional significance of local circadian clocks in maintaining oral immune homeostasis.

From a mechanistic perspective, future research should progress beyond linear pathway analysis toward a systems-level understanding of circadian–immune networks. Classical studies have often examined inflammatory pathways such as NF-κB, NLRP3, MAPK, and JAK/STAT independently; however, within physiological contexts, these signaling modules are highly interconnected and coordinated by core clock machinery through inflammatory transcriptional regulation, metabolic reprogramming, and oxidative stress responses. Systems biology approaches integrating transcriptomics, proteomics, metabolomics, and microbiome profiling will therefore be essential to decipher how circadian networks regulate inflammatory amplitude, persistence, and tissue repair processes.

Targeting the molecular clock to modulate inflammatory diseases has emerged as an attractive therapeutic strategy, but clinical translation requires careful evaluation. Pharmacological regulators of REV-ERB and ROR pathways have been widely used to investigate the relationship between circadian regulation and inflammation. However, several commonly used compounds have important limitations. For example, although SR9009 and SR8278 are frequently employed as REV-ERB modulators, emerging evidence indicates potential REV-ERB-independent effects, suggesting that their biological activities should not be attributed exclusively to clock regulation. Moreover, SR1078 primarily influences BMAL1 expression through activation of RORα/γ signaling rather than acting as a direct BMAL1 agonist. Therefore, future studies investigating circadian-targeting drugs should incorporate genetic deletion models, genome editing approaches, and independent validation strategies to avoid misinterpretation caused by pharmacological off-target effects.

From a translational perspective, chronotherapy provides a promising framework for developing precision strategies in oral disease management. Many oral diseases exhibit pronounced temporal characteristics, including rhythmic variations in salivary secretion, microbial metabolic activity, and inflammatory responses. Consequently, the timing of therapeutic intervention may influence treatment efficacy and tissue regenerative capacity. However, chronotherapeutic approaches in oral medicine remain largely conceptual, with limited clinical validation. Future studies integrating individual circadian profiles, salivary biomarkers, and microbial rhythmic signatures may facilitate the development of personalized temporal medicine strategies for oral diseases.

Overall, circadian biology provides a novel conceptual framework for understanding oral immune homeostasis and disease pathogenesis. By integrating chronobiology, immunology, microbiome science, and systems medicine, future research may uncover the temporal codes governing oral disease progression and establish innovative rhythm-based diagnostic and therapeutic approaches. A growing body of evidence indicates that the circadian clock serves as a critical regulator of immune function, inflammatory networks, and microbial ecosystem dynamics. Nevertheless, mechanistic validation and direct human evidence in oral tissues remain insufficient. Future investigations should integrate temporal biology into oral research frameworks by considering the coordinated interactions among host rhythms, immune regulation, microbial ecology, and behavioral factors. Establishing such an integrated model of oral chronomedicine may uncover previously unrecognized mechanisms of oral disease pathogenesis and enable the development of personalized preventive and therapeutic strategies based on individual circadian profiles.
