# Correction: The oral immune and inflammatory network under circadian clock control

**DOI:** 10.3389/fimmu.2026.1992894

**Published:** 2026-10-06

**Authors:** Xuping Sun, Xiaoxue Ma, Yunjing Ma, Xuezhen Wang, Xiaoguang Li, Mei Ji

**Affiliations:** 1 Department of Stomatology, Shandong Provincial Hospital Affiliated to Shandong First Medical University, Jinan, Shandong, China; 2 Shandong First Medical University, Jinan, Shandong, China; 3 Department of Stomatology, Shizhong District People’s Hospital of Jinan, Jinan, Shandong, China

**Keywords:** circadian rhythm, clock genes, immune and inflammatory diseases, oral microbiome, oral tissues

Affiliation 1 was erroneously given as “Department of Stomatology, Shandong Provincial Hospital Affiliated to Shandong First Medical University, Shandong First Medical University, Jinan, Shandong, China” which combined “Shandong Provincial Hospital Affiliated to Shandong First Medical University” and “Shandong First Medical University” into a single affiliation. It has been corrected now as: Aff 1 “1 Department of Stomatology, Shandong Provincial Hospital Affiliated to Shandong First Medical University, Jinan, Shandong, China” and Aff 2: 2 Shandong First Medical University, Jinan, Shandong, China”.

Also, Aff 3: “Department of Stomatology, Shandong Provincial Hospital Affiliated to Shandong First Medical University, Jinan, Shandong, China” was same as Aff 1, So, this has been removed.

As a result, the remaining affiliations were renumbered and have been correctly assigned to the authors.

The original version of this article has been updated.

